# A Narrative Review of Artificial Intelligence in MRI-Guided Prostate Cancer Diagnosis: Addressing Key Challenges

**DOI:** 10.3390/diagnostics15111342

**Published:** 2025-05-26

**Authors:** Deniz Alis, Aslihan Onay, Evrim Colak, Ercan Karaarslan, Baris Bakir

**Affiliations:** 1 Department of Radiology, School of Medicine, Acibadem Mehmet Ali Aydinlar University, 34750 Istanbul, Atasehir, Turkey; deniz.alis@acibadem.edu.tr (D.A.); ercan.karaarslan@acibadem.edu.tr (E.K.); 2 Department of Radiology, Faculty of Medicine, TOBB University of Economics and Technology, Beştepe Mah Yasam Cad No. 5, 06510 Ankara, Yenimahalle, Turkey; aslionay@gmail.com; 3 Electrical and Electronics Engineering Department, Ankara University, 50. Yil Yerleskesi Bahcelievler Mah, 06830 Ankara, Golbasi, Turkey; 4 Turkish Accelerator and Radiation Laboratory (TARLA), Ankara University, 50. Yil Yerleskesi Bahcelievler Mah, 06830 Ankara, Golbasi, Turkey; 5 Department of Radiology, Istanbul Faculty of Medicine, Istanbul University Capa, 34093 Istanbul, Fatih, Turkey; drbarisbakir@yahoo.com

**Keywords:** magnetic resonance imaging (MRI) of prostate, artificial intelligence (AI), machine learning (ML), deep learning (DL), prostate cancer (PCa)

## Abstract

**Background/Objectives**: Magnetic resonance imaging (MRI) is crucial in detecting suspicious lesions and diagnosing clinically significant prostate cancer (csPCa). However, variability in MRI-targeted diagnostic pathways arises due to factors such as patient characteristics, imaging protocols, and radiologist expertise. Artificial intelligence (AI) offers potential solutions to these challenges by enhancing diagnostic accuracy and efficiency. **Methods**: This narrative review explores AI techniques, particularly machine learning and deep learning, in the context of prostate cancer diagnosis. It examines their application in improving MRI scan quality, detecting artifacts, and assisting radiologists in lesion detection and interpretation. It also considers how AI helps to reduce reading time and inter-reader variability. **Results**: AI has demonstrated sensitivity that is generally comparable to experienced radiologists, although specificity tends to be lower, potentially increasing false-positive rates. The clinical impact of these results requires validation in larger, prospective multicenter studies. AI is effective in identifying artifacts, assessing MRI quality, and assisting in diagnostic efficiency by providing second opinions and automating lesion detection. However, variability in study methodologies, datasets, and imaging protocols can impact AI’s generalizability, limiting its broader clinical application. **Conclusions**: While AI shows significant promise in enhancing diagnostic accuracy and efficiency for csPCa detection, challenges remain, particularly with the generalizability of AI models. To improve AI robustness and integration into clinical practice, multicenter datasets and transparent reporting are essential. Further development, validation, and standardization are required for AI’s widespread clinical adoption.

## 1. Introduction

Prostate cancer (PCa) is the second most common cancer in men, with about 1.4 million new cases annually. Its broad genomic diversity leads to varied patient outcomes [1]. Indolent PCa is common, progresses slowly, and has a low cancer-related mortality rate [2,3,4,5]. On the other hand, clinically significant prostate cancer (csPCa) is linked to rapid progression, higher mortality, and increased morbidity risks [3,6]. The early detection of csPCa is essential, although distinguishing it from clinically insignificant cancer remains an ongoing challenge.

Performing a prostate MRI before biopsy can reduce unnecessary procedures, overdiagnosis, and overtreatment while improving csPCa detection [7,8,9]. As a result, the European Association of Urology (EAU), the National Institute for Health and Care Excellence (NICE), and the American Urological Association (AUA) have incorporated multi-parametric MRI (mpMRI) into their clinical guidelines as a key element of PCa diagnosis [10,11,12].

Prostate MRI helps confirm or rule out csPCa, but its performance varies. Specifically, the positive predictive value (PPV) for MRI can be affected by false-positive rates, which may reach up to 50% [13]. Standardizing MRI acquisition, interpretation, prostate imaging reporting and data system (PI-RADS) scoring, and reporting is essential for improving diagnostic accuracy [13,14,15].

Patient motion artifacts, rectal gas, body habitus, and hip prostheses have been reported to negatively affect MRI quality [16]. Additionally, variations in MRI equipment, such as differences in magnet strength, coil quality, and pulse sequence parameters, can further disrupt image quality [17]. To mitigate these challenges, the prostate imaging quality (PI-QUAL) scoring system was created to standardize the assessment of image quality [18,19].

**Figure 1:**
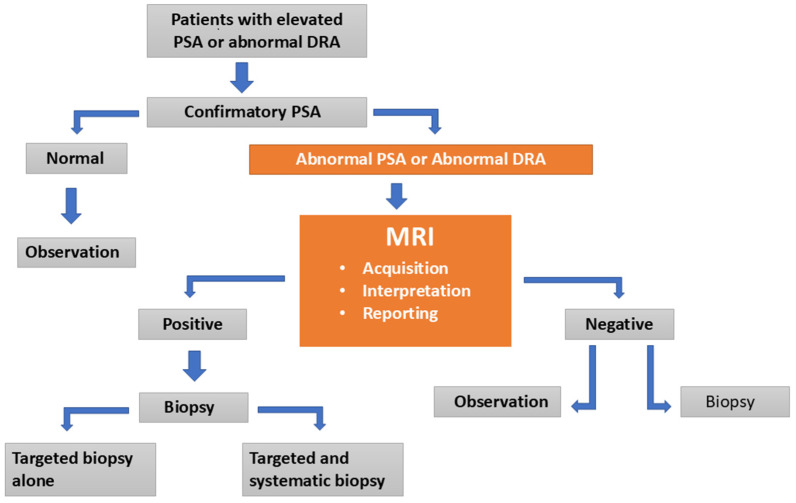
Despite advancements in multi-parametric magnetic resonance imaging (mpMRI) technology, significant variability persists in magnetic resonance imaging (MRI) acquisition, interpretation, prostate imaging and reporting system (PI-RADS) scoring, and reporting, which can affect diagnostic accuracy. The MRI-first approach, recommended before biopsy in men with suspected clinically significant prostate cancer (csPCa), aims to improve early detection and reduce unnecessary biopsies. Artificial intelligence (AI) holds considerable potential to address this variability and contributes to the standardization of critical aspects such as MRI acquisition, interpretation, and reporting. Within the overall diagnostic pathway for prostate cancer (PCa) detection, orange-colored boxes represent the role of MRI guidance.

The PI-RADS was introduced in 2012, updated in 2015 (PI-RADS V2), and revised in 2019 (PI-RADS V2.1) to standardize both prostate MRI acquisition and interpretation [17,19,20]. PI-RADS is a scoring system ranging from 1 to 5, used to assess the likelihood of PCa based on observed MRI abnormalities. While PI-RADS has helped to standardize report quality and improve interdisciplinary communication, it also has limitations, including moderate specificity in detecting csPCa [14]. Furthermore, studies have shown that the accuracy of csPCa detection is significantly influenced by the interpreting radiologists’ expertise, resulting in considerable inter-reader variability [14]. Benign lesions such as benign prostatic hyperplasia, prostatitis, or atrophy may be incorrectly identified as PCa, leading to unnecessary biopsies, while some cases of csPCa may be overlooked by even experienced radiologists due to their resemblance to benign conditions or insufficient visibility on MRI [21]. The MR-targeted diagnostic pathway of PCa is illustrated in Figure 1.

Amid these challenges, artificial intelligence (AI) technologies have emerged and are being increasingly integrated into medical workflows to enhance image acquisition, analysis, and interpretation, thereby improving diagnostic performance. In a paired multi-center and multi-reader retrospective confirmatory study, an AI-based system outperformed radiologists in detecting Gleason grade group 2 or higher cancers, with area under the receiver operating characteristic curve (AUROC) values of 0.86 for radiologists and 0.91 for the AI system [22]. However, these promising results should be cautiously interpreted due to their retrospective nature, and validation through prospective multicenter trials remains essential.

AI has significant potential for clinicians and can play a crucial role in primary diagnostics. It can optimize MRI workflows by improving image quality and aiding clinical interpretation, while also reducing inter-reader variability. From the patient’s perspective, AI can help avoid unnecessary biopsies and ensure access to more accurate diagnoses.

In this review, the contribution of AI in addressing key challenges in MRI-guided PCa diagnostics is explored. With a specific focus on the prebiopsy phase, incorporation of AI in (1) standardizing and improving MRI quality, (2) enhancing csPCa detection while minimizing unnecessary interventions, and (3) reducing inter-reader variability will be examined.

### Literature Search

This narrative review synthesizes the recent literature on AI applications in MRI-guided diagnosis of csPCa. To ensure methodological transparency and reproducibility, we employed a structured literature search strategy using two primary medical databases.

Databases Searched: PubMed, Scopus

Search Period: January 2010–February 2025

Search Terms (keywords and combinations): Prostate cancer (PCa, csPCa); Magnetic resonance imaging (MRI, multi-parametric MRI, mpMRI, bi-parametric MRI, bpMRI); Artificial intelligence (AI, machine learning, ML, deep learning, DL, radiomics); Diagnosis (lesion detection, image quality, PI-RADS, interpretation variability).

Inclusion Criteria:

1. Peer-reviewed full-text articles in English.
2. Human studies involving participants diagnosed or clinically suspected of having PCa/csPCa.
3. Studies utilizing mpMRI or bi-parametric MRI (bpMRI).
4. Studies confirming csPCa through pathological reference standards (biopsy or radical prostatectomy).
5. Articles explicitly focused on AI applications in MRI-guided PCa diagnosis, specifically covering: AI-driven enhancements in MRI acquisition (image quality and acquisition time); AI-based assessment and standardization of MRI image quality; AI performance metrics in lesion detection, PI-RADS scoring, diagnostic accuracy, and radiologist comparisons; and AI-assisted reduction in inter-reader variability.

Exclusion Criteria:

1. Non-human studies (animal models, phantoms).
2. Conference abstracts, commentaries, editorials, letters, case reports, or articles lacking primary data.
3. Articles without direct clinical relevance or those unavailable in full text.
4. Studies not explicitly aligned with the predefined thematic focus of this review.

Following initial database searches, reference lists from included studies were meticulously reviewed to identify additional relevant articles. Each selected article underwent critical analysis and was categorized according to the core themes listed above, with particular attention given to articles from reputable, high-impact journals, and those demonstrating methodological rigor or significant innovation in AI implementation within prostate MRI workflows.

Given the narrative approach of this review—rather than a systematic review—we have not included a PRISMA-style flowchart. However, the detailed description above ensures transparency regarding our literature selection methodology, consistent with high-quality narrative review standards.

## 2. AI in Prostate MRI Acquisition and Interpretation

### 2.1. The Potential of AI in Prostate MRI: Advancements and Opportunities

Tasks typically associated with human intelligence, such as decision-making, learning, and problem-solving, can be performed by AI systems after undergoing a specific training process. However, large datasets are required to train algorithms for such tasks in machine learning (ML), a subset of AI. Within ML, algorithms can be categorized as supervised, where training occurs on labeled data, or unsupervised, which allows the AI system to identify hidden patterns in unlabeled data. In this context, several studies have reported the use of AI in prostate segmentation, intraprostatic lesion detection, and classification, indicating that AI can be effectively integrated into the prostate MRI workflow [23].

Deep Learning (DL), an advanced branch of ML, utilizes Convolutional Neural Networks (CNNs) and has demonstrated outstanding performance in medical image analysis, including disease classification, tumor detection, and anatomical segmentation [24,25,26,27,28]. Furthermore, Recurrent Neural Networks (RNNs) are well-suited for processing dynamic imaging data, such as time-series MRI scans [29], while Generative Adversarial Networks (GANs) generate synthetic data to enhance model training and improve accuracy [30].

**Figure 2:**
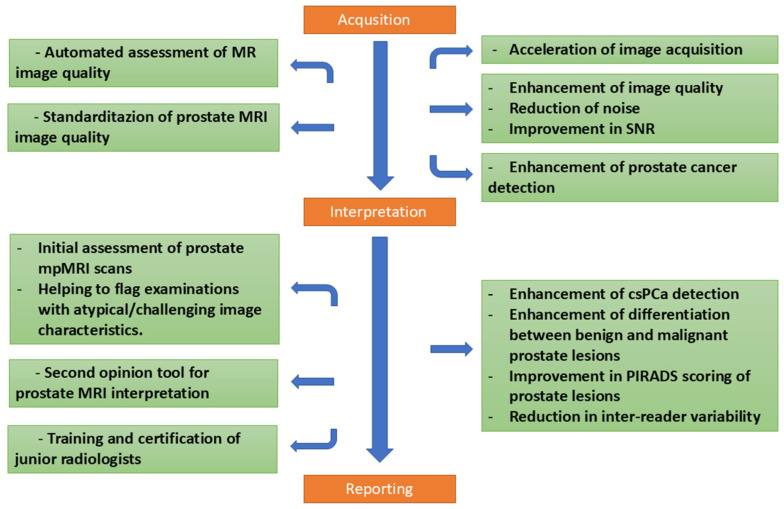
AI-based methods have demonstrated promising potential to enhance multiple stages of the MRI-guided prostate cancer PCa diagnostic pathway. The figure illustrates AI′s role across three main components: (1) MRI Acquisition, where AI contributes through quality assurance, artifact detection, and deep-learning-based image reconstruction to improve image clarity and reduce acquisition times; (2) MRI Interpretation, in which AI provides automated lesion detection, segmentation, classification, and serves as a second-opinion tool to reduce inter-reader variability; and (3) Reporting, highlighting AI’s capacity to standardize results, provide structured lesion quantification, and facilitate clear communication of imaging findings. Collectively, these applications underscore AI’s potential to standardize and optimize the diagnostic process in clinical practice. Orange-colored boxes indicate the key stages of prostate MRI processing—namely acquisition, interpretation, and reporting. The green boxes branching from them reflect clinical challenges associated with each stage, along with the contributions and potential solutions provided by AI.

AI has great potential in medical image acquisition and interpretation. It can enhance image quality by reducing noise, correcting artifacts, and improving resolution—critical for accurate diagnoses. AI also enables the automation of medical image analysis, ensuring consistent and reliable results. Importantly, AI can help reduce inter-reader variability by providing standardized interpretations, thus improving the consistency and reliability of diagnostic processes. The overview of potential contributions of AI to the PCa diagnostic pathway are schematized in Figure 2.

### 2.2. AI and Enhancing Image Quality and Reducing Acquisition Time

To evaluate distinct clinical parameters, four key imaging sequences are defined for prostate MRI according to the PI-RADS guidelines: T1-weighted imaging (T1WI), T2-weighted imaging (T2WI), diffusion-weighted imaging (DWI), and dynamic contrast-enhanced (DCE) imaging. The PI-RADS V2.1 guidelines [17] outline specific attributes for each sequence, ensuring the accuracy and reproducibility of prostate MRI findings by setting minimum parameter criteria for these sequences Table 1.

AI integration in prostate MRI scans leads to significantly reduced acquisition times compared to traditional MRI techniques, while also improving image quality. This enhancement is made possible by DL reconstruction algorithms, which effectively reduce noise and minimize Gibbs ringing artifacts [31].

The longest portion of MRI acquisition time is typically dedicated to T2WI and DWI sequences. Therefore, efforts to reduce MRI acquisition time have primarily targeted shortening the duration of these two sequences. In contrast, T1WI sequences require relatively short acquisition times. Additionally, the acquisition duration of DCE sequences is influenced by the physiological contrast agent wash-in time, which cannot be externally controlled [31]. Furthermore, in a recent study, Huang et al. developed a DL model (pix2pix algorithm) to synthesize DCE-MRI sequences from non-contrast MRI, including T1WI, T2WI, DWI, and apparent diffusion coefficient (ADC) maps. The simulated DCE-MRI showed high similarity to the original sequences, and there was excellent agreement with radiologists’ PI-RADS scores. Notably, the addition of simulated DCE-MRI to bpMRI resulted in 34 out of 323 patients being upgraded from PI-RADS 3 to PI-RADS 4 [32].

AI accelerates mpMRI by reducing signal averages, undersampling *k*-space, and optimizing acquisition parameters. These techniques shorten scan times while preserving image quality. Additionally, increasing the parallel imaging factor or acquiring fewer *b*-values are alternative approaches that can further expedite the imaging process. Moreover, the use of AI for the synthesis of high *b*-value images or ADC maps can yield faster DWI scans. These combined approaches have the potential to improve scan efficiency while diagnostic accuracy is still maintained [31]. These advanced algorithms contribute in two ways: (i) by reducing image acquisition times and (ii) by reducing motion artifacts, which are common in MRI scans. As a result, AI techniques enhance signal-to-noise ratio (SNR) and image sharpness, resulting in higher-quality images [31]. Image reconstruction via DL can also improve image quality. In one retrospective study involving 30 patients, T2WI images which are reconstructed by DL outperformed conventional MRI in lesion detectability, diagnostic confidence, and overall image quality [33]. Another retrospective study involving 84 patients whose images are evaluated by two radiologists, found that complex-averaged calculated high *b*-values offered higher diagnostic accuracy for detecting significant prostate lesions in comparison to magnitude-averaged acquired high *b*-values [34].

An AI-based acceleration of the mpMRI protocol can be achieved through various strategies, such as reducing signal averages, undersampling *k*-space, and optimizing acquisition parameters. Increasing the parallel imaging factor or acquiring fewer *b*-values are additional methods to further speed up the imaging process. Furthermore, using AI to synthesize high *b*-value images or ADC maps can accelerate DWI scans. These combined approaches can enhance scan efficiency while maintaining diagnostic accuracy [31]. These advanced algorithms improve the process in two ways: (i) by reducing image acquisition times and (ii) by minimizing motion artifacts, which are common in MRI scans. As a result, AI techniques enhance SNR and image sharpness, leading to higher-quality images [31]. DL image reconstruction can also improve image quality. In a retrospective study with 30 patients, DL-reconstructed T2WI images outperformed conventional MRI in lesion detectability, diagnostic confidence, and overall image quality [33]. Another retrospective study, involving 84 patients and evaluated by two radiologists, found that complex-averaged high *b*-values provided higher diagnostic accuracy for detecting significant prostate lesions compared to magnitude-averaged acquired high *b*-values [34]. Ueda et al. demonstrated that DL-reconstructed DWI images with *b*-values ranging from 1000 s/mm^2^ to 5000 s/mm^2^ exhibited significantly higher SNR and CNR compared to non-DL-reconstructed DWI images [35]. In contrast, some studies have shown that although DL-reconstructed images enhance image quality, lesion detection rates remain comparable to those obtained with conventional MRI [36,37]. For instance, in this larger study involving 155 patients, DL-reconstructed 2D turbo spin echo (TSE) images demonstrated superior SNR compared to conventional images, yet lesion detection rates were similar [37]. Another retrospective study of 124 patients compared extrapolated high *b*-value images, generated from lower *b*-values, with conventionally acquired high *b*-value DWI. The results showed that the extrapolated images provided better background signal suppression, improved lesion visibility, and reduced distortion, while maintaining comparable tumor detection rates [38]. In fact, some studies have found no significant differences in image quality between the two modalities [37,39,40]. A retrospective study involving 46 patients revealed that DL-reconstructed images, despite shorter acquisition times, were of comparable quality to conventional T2WI [41].

### 2.3. AI and Image Quality Assessment

Prostate MRI is essential for diagnosing PCa. As such, MRI image quality can significantly influence the entire diagnostic process. The PI-QUAL scoring system, initially derived from the “prostate evaluation for clinically important disease: sampling using image guidance or not?” (PRECISION) trial [7,18], was developed by European prostate MRI experts to assess the diagnostic quality of prostate MR images. PI-QUAL V1 is based on a scale from 1 to 5, evaluating T2WI, DWI, ADC, and DCE images according to minimal technical parameters outlined in PI-RADS recommendations, along with considerations for anatomical landmarks and artifacts [18]. PI-QUAL V1 studies show that image quality directly affects csPCa detection and biopsy decisions [42,43]. However, PI-QUAL V1 has certain limitations. It includes bpMRI protocols that do not use intravenous (IV) contrast agents and has limited applicability in clinical situations beyond biopsy-related decisions, such as tumor staging, active surveillance, population screening, and follow-up of patients with prior scans [19]. To address these limitations, the European Society of Urogenital Radiology (ESUR) Prostate Cancer Working Group developed PI-QUAL V2, which simplifies the scoring system to a 3-point scale and is also adapted for prostate MRI examinations that utilize IV contrast agents [19].

Inter-reader variability in PI-QUAL V1 is another aspect that requires further investigation. Giganti et al. reported excellent inter-reader agreement between two experienced readers for each PI-QUAL score, emphasizing the reliability of the system [44]. Similarly, Basar et al. demonstrated good inter-reader agreement across various MRI sequences in a multicenter setting [45]. However, Fleming et al. noted that while PI-QUAL V1 showed good reproducibility, variability was observed in lower-quality images. It was suggested that the accuracy of the system might be influenced by the image quality being evaluated [46].

In clinical settings, PI-QUAL V2 has shown to be moderately reliable for assessing prostate MRI image quality among readers with varying expertise [47]. As a recent update, its full potential in image quality assessment will become clearer through further research. However, it is evident that image quality assessment is pivotal in the standardization of prostate MRI interpretation. In this regard, Coelho et al. suggested that the PI-QUAL score be incorporated as a standard image quality metric in radiology reports [48]. The adoption of semi-automated or automated approaches for prostate image quality assessment is expected to streamline radiology workflows while also contributing to the standardization of prostate MRI interpretation.

AI holds significant potential to assist in the automated assessment of prostate MRI quality, facilitating real-time quality control. AI-based systems capable of real-time quality assessment are already widely used in non-medical fields, but studies in medical imaging remain limited. For instance, Cipollari et al. demonstrated that CNNs could classify prostate MRI images at the individual slice level with high accuracy, and at the entire sequence level with near-perfect performance [49]. Similarly, Yue Lin et al. showed that a DL-based AI model achieved performance comparable to expert radiologists in classifying the image quality of high-resolution T2WI prostate MRI images obtained from datasets with varying quality. Furthermore, the study revealed that high-quality T2WI led to higher detection rates of csPCa in targeted biopsies [50] In addition, in another study by Lin et al., it was demonstrated that a DL-based AI algorithm for prostate MR image quality classification effectively predicted extraprostatic extension (EPE) at final pathology, especially when higher-quality T2WI images were available [51].

In summary, recent advancements in prostate MRI, driven by AI, have focused on reducing acquisition times, enhancing image quality, and streamlining image quality assessment. AI techniques have accelerated MRI protocols, resulting in shorter scan durations without compromising diagnostic accuracy. The application of DL algorithms has been particularly successful in reconstructing images with improved SNR and contrast, which in turn increases lesion detectability.

AI is also recognized for its potential in automating the assessment of MRI image quality, enabling real-time quality control and standardizing image evaluation. However, challenges remain, including variability in AI performance and the need for large, diverse datasets. Despite these obstacles, as AI continues to be integrated into clinical workflows, it is expected to improve consistency in image quality assessment and ultimately enhance patient care. Furthermore, AI’s increasing prominence in the PCadiagnostic landscape suggests that its applications will go beyond improving image quality and acquisition time. AI’s potential to enhance diagnostic accuracy is particularly evident in the interpretation of prostate MRI scans, addressing both inter- and intra-reader variability. This can significantly aid radiologists in lesion detection and classification, as the success of MRI-guided biopsies heavily depends on precise imaging data interpretation. The following section will explore the role of AI in improving prostate MRI scan interpretation, its current limitations, and its future potential in clinical practice.

### 2.4. Prostate MRI Interpretation and the Role of AI

The introduction of mpMRI has greatly enhanced PCa detection, shifting from traditional transrectal ultrasound (TRUS)-guided biopsies to MRI-targeted methods. While it has moderate specificity, numerous large-scale studies have shown that MRI-targeted biopsies outperform in detecting csPCa, providing high sensitivity [7,8,9]. This diagnostic method requires team effort, involving MR technologists, radiologists, urologists, interventional radiologists, and pathologists. Key factors for success include obtaining high-quality MR images, precise image interpretation, accurate biopsy guidance, and thorough pathological analysis for diagnosis and Gleason grading [52], with AI offering the potential for significant improvements.

PCa detection accuracy depends on the radiologist’s expertise in interpreting MRI scans [14,53,54]. Despite progress in mpMRI technology, there remains notable variability in both inter- and intra-reader results, which can lead to diagnostic inconsistencies [14,15]. In this regard, radiologist expertise plays a pivotal role in detection accuracy, underscoring the need for standardized training and certification to ensure more consistent interpretations [55]. To achieve uniformity in prostate MRI interpretation across radiologists, standardized training programs and certification processes are essential, ultimately improving patient care and diagnostic accuracy [56]. These challenges in the PCa diagnostic process can be mitigated with AI, which can also assist in enhancing detection and classification by distinguishing between benign and malignant lesions.

AI is valuable for the initial assessment of prostate mpMRI scans. It can flag examinations with atypical or challenging image characteristics. Furthermore, AI tools can serve as second-opinion systems, aiding in lesion detection and classification, which helps reduce inter-reader variability [57]. AI-powered models can also play a pivotal role in the training and certification of radiologists by providing real-time feedback and pinpointing areas for improvement in image interpretation [57].

Despite the robustness issues encountered in the recent past [23,58], a progress towards incorporation of unsupervised AI methods for clinical use is also evident [59,60,61,62]. AI models for intraprostatic lesion detection remain largely experimental, with limited integration into routine clinical practice [63,64,65]. However, as research progresses, AI-driven models—particularly those trained on diverse datasets that incorporate various imaging techniques, patient demographics, and clinical contexts—hold the potential to standardize prostate MRI interpretation and improve diagnostic outcomes. In the future, AI may play an increasingly central role in enhancing diagnostic accuracy, efficiency, and accessibility in PCa care.

**Figure 3:**
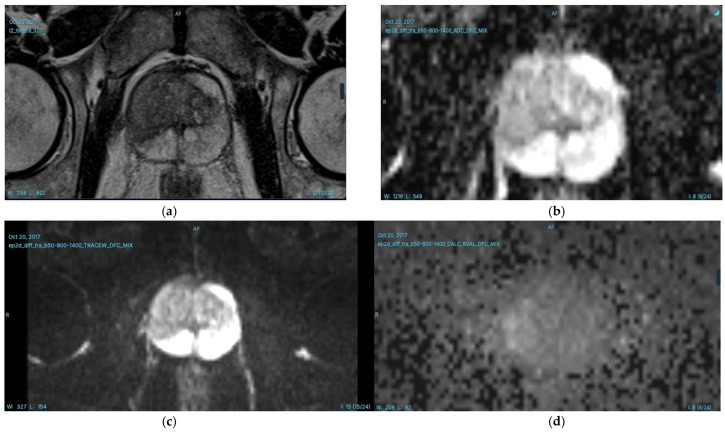
T2WI (**a**) ADC map (**b**) DWI) (**c**) and calculated *b*1500 DWI (*d*) images of a 63-year-old man with a serum prostate-specific antigen (PSA) level of 3.7 ng/mL. A focal hypointense prostate lesion is identified in the right peripheral zone (PZ), measuring 28 mm in diameter. The lesion is markedly hypointense on the ADC map and slightly hyperintense on high *b*-value images. According to PI-RADS v2.1 criteria, the lesion is classified as PI-RADS 3. Additionally, another PI-RADS 3 lesion is observed in the left lateral PZ, measuring 14 mm in diameter. This lesion appears focal hypointense on T2WI, markedly hypointense on the ADC map, and is indistinguishable on high *b*-value diffusion-weighted images.

**Figure 4:**
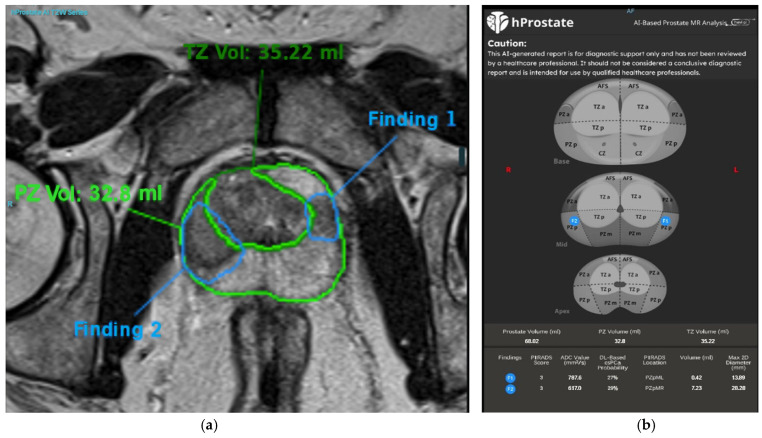
T2WI images (**a**) and a prostate sector map (**b**) showing marked prostate lesions in a 63-year-old man with a serum PSA level of 3.7 ng/mL (same patient as in Figure 3). The deep learning (DL)-based application has segmented prostate lesions in the right and left lateral PZs, assigned PI-RADS scores, and determined the dimensions and volume of the lesions. The lesion locations are also marked on the prostate sector map for potential guidance in MRI-targeted biopsy. This lesion was clinically diagnosed as focal prostatitis, and the patient was scheduled for follow-up. In this case, AI provided the necessary prostate lesion assignments in advance, potentially reducing reading time and assisting less-experienced readers in detecting lesions and assigning PI-RADS scores. This is particularly valuable because DL models are trained on expert-annotated datasets, effectively transferring the knowledge of expert prostate imaging readers to less-experienced practitioners. By doing so, AI contributes to democratizing diagnostic imaging in a scalable manner.

**Figure 5:**
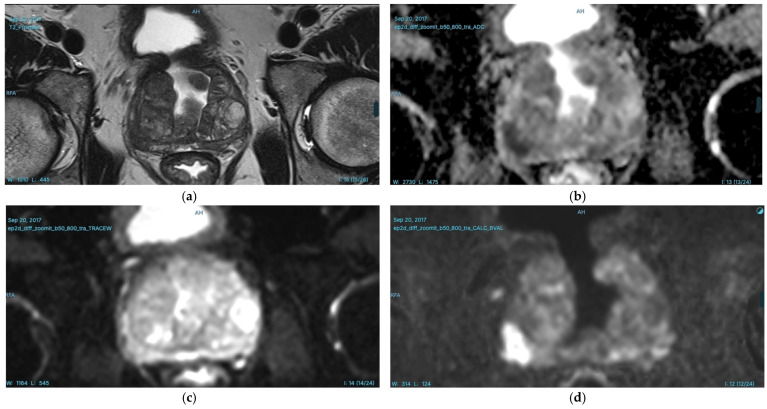
T2WI (**a**), ADC map (**b**), DWI (**c**), and calculated *b*1500 DWI (**d**) images of a 65-year-old man with a serum PSA level of 6.4 ng/mL. A focal hypointense prostate lesion measuring 18 mm in diameter is identified in the right PZ, appearing markedly hypointense on the ADC map and markedly hyperintense on high *b*-value images. Additionally, another prostate lesion measuring 17 mm in diameter is observed in the left posterolateral PZ, appearing focally hypointense on T2WI, markedly hypointense on the ADC map, and markedly hyperintense on high *b*-value diffusion-weighted images. According to PI-RADS v2.1 criteria, both prostate lesions are classified as PI-RADS 5. Targeted biopsy confirmed csPCa. AI-assisted prostate MRI interpretation can play a crucial role in detecting csPCa, improving risk stratification, and guiding biopsy decisions. In this case, AI could have assisted in the following: enhancing lesion detection and segmentation, ensuring both lesions were identified and properly characterized; standardizing PI-RADS assessment, reducing inter-reader variability and increasing diagnostic confidence; supporting MRI-targeted biopsy planning by accurately marking lesion locations on a prostate sector map, improving sampling precision; optimizing workflow efficiency by reducing reading time and providing quantitative lesion metrics, which may help in csPCa risk assessment and treatment planning. By leveraging AI in prostate MRI evaluation, radiologists can enhance diagnostic accuracy, minimize missed csPCa cases, and support less-experienced readers in high-stakes clinical decision-making.

**Figure 6:**
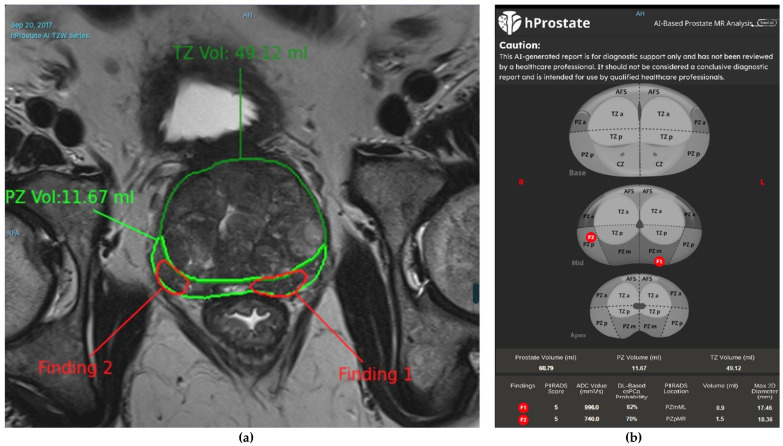
T2WI images (**a**) and prostate sector map (**b**) showing marked prostate lesions in a 65-year-old man with a serum PSA level of 6.4 ng/mL (same patient as in Figure 5). The DL-based application has segmented prostate lesions in the right lateral PZ and left posterolateral PZ, assigned PI-RADS scores, and determined the dimensions and volume of the prostate lesions. The lesion locations are also marked on the prostate sector map for guidance in MR-targeted biopsy.

In our radiology clinic, a DL-based application is used for PCa detection. Integrated within the radiology workflow through the picture archiving and communication system (PACS), this application detects and segments suspicious prostate lesions while also performing PI-RADS scoring. Additionally, suspicious lesions are highlighted on the prostate gland sector map, providing size and volume information, which significantly contributes to biopsy guidance. An example of PI-RADS 3 lesion is given in Figure 3. In our clinical practice, we observe the potential of this technology and aim to contribute to its further development, which will enable the accurate diagnosis of more patients in the future [66]. Consequently, DL-based segmentation of the same lesion is presented in Figure 4. Another set of images of a different patient who has two PI-RADS 5 lesions is shown in Figure 5. Results for the segmentation of lesions obtained by DL techniques of this second patient are given in Figure 6.

### 2.5. Approaches to AI-Based Detection of PCa

AI-based approaches to detecting PCa primarily fall into two categories: ML and DL. Both techniques have shown promise in differentiating csPCa from benign conditions, each with its strengths and limitations.

#### 2.5.1. ML Approaches

ML, a prominent subfield of AI, involves algorithms capable of learning patterns from data to facilitate predictions without explicit task-specific programming. ML techniques have increasingly been adopted in medical imaging, particularly for (csPCa) detection, where they assist radiologists by identifying subtle imaging features indicative of disease.

One prevalent ML method is radiomics, which entails extracting quantitative features from medical images to characterize tumor properties such as shape, texture, and intensity. Radiomics can capture tumor characteristics that may not be visually discernible to radiologists, thereby aiding differentiation between csPCa and indolent lesions [67,68,69]. Radiomics-based ML workflows typically consist of two critical phases:

Feature Extraction:

This step involves the systematic extraction and quantitative measurement of specific lesion characteristics, focusing on morphological features (e.g., size, shape), textural attributes including those derived from the Gray Level Co-occurrence Matrix (GLCM)—a statistical method that evaluates the spatial relationship between pixel intensities (e.g., roughness, homogeneity, contrast, correlation, energy, entropy), and intensity distributions (e.g., brightness variations). These methods aim to quantitatively characterize subtle disease-related patterns that may not be detectable through conventional visual inspection. The extracted features are then quantitatively encoded as numerical descriptors, facilitating subsequent computational analysis and interpretation.

Classification: Extracted radiomic features are subsequently analyzed by ML algorithms, such as Support Vector Machines (SVMs), Random Forests, or Gradient Boosting Machines. These models classify lesions by comparing extracted features against established patterns derived from known benign or malignant cases [67,68,69].

Despite the demonstrated potential of radiomics-based ML models in csPCa detection and risk stratification, several challenges persist. Primarily, these methods require extensive human input during feature engineering, where domain experts must select the most clinically relevant features. This subjective process introduces variability and limits reproducibility across different studies and institutions. Moreover, the accuracy and effectiveness of radiomics approaches are sensitive to preprocessing procedures, including lesion segmentation and standardized feature selection, further complicating broader clinical implementation [68].

#### 2.5.2. DL Approaches

DL, a specialized subfield of ML, eliminates the necessity for manual feature selection by enabling models to learn essential patterns directly from medical imaging data. Among DL techniques, CNNs [70] have become particularly influential in radiology, significantly advancing the interpretation of prostate MRI by automatically identifying and classifying suspicious lesions with notable accuracy.

The growing interest in DL in recent years can be largely attributed to the availability of large datasets, advancements in model architectures, and the accessibility of powerful and affordable computing hardware, particularly graphic processing units (GPUs). GPUs, with their parallel processing capabilities, have become indispensable in training complex DL models, drastically reducing the time required for computations and enabling real-time analysis of large medical datasets [71]

In the context of PCa detection, the need for efficient and rapid processing of high-resolution medical images is paramount. DL models, as a form of representation learning, automatically extract and learn hierarchical features that reflect the intricate structures of medical data, such as MRI and histopathological scans. The integration of GPUs has not only made these processes feasible but has also accelerated the development of highly accurate models for tasks like early PCa detection, improving diagnostic accuracy and supporting clinical decision-making [72].

In contrast to traditional ML methods, which depend heavily on manually extracted radiomic features, CNNs analyze MRI images by processing raw pixel-level data. CNNs operate through multiple interconnected layers of artificial neurons, each capable of recognizing progressively complex patterns. The initial layers typically detect basic features such as edges and shapes, intermediate layers capture textures and delineate tumor boundaries, and deeper layers identify high-level characteristics, including the likelihood of a lesion being clinically significant [65]. 

The ability of CNNs to automatically learn optimal features from imaging data makes them particularly advantageous for csPCa detection. Unlike radiomics, which depends on human-defined features and thus may not fully capture all relevant data patterns, CNN-based models autonomously identify meaningful features, enhancing their adaptability and potentially their diagnostic accuracy. Despite these advantages, several challenges exist in the application of DL models. Chief among these is the requirement for large, diverse, and meticulously annotated datasets to train robust CNNs effectively. The quality and diversity of available training data critically influence the generalizability and reliability of these models. Moreover, variability in clinical imaging protocols, patient populations, and MRI scanner types can impact DL model performance in practical settings [64].

Despite these ongoing challenges, DL methodologies, especially CNNs, are expected to increasingly underpin csPCa diagnostics. Advances in transfer learning and data augmentation techniques offer potential pathways to enhance model scalability, reduce human oversight, and ensure diagnostic consistency across varied clinical contexts [64]. Furthermore, hybrid models that combine radiomic features, clinical data, and DL approaches have recently demonstrated superior performance compared to individual modalities [73,74].

For instance, the study by Chen et al. introduced a multimodal DL nomogram that integrates clinical variables, PI-RADS scores, and radiomic features derived from bpMRI to predict csPCa in patients with gray-zone prostate-specific antigen (PSA) levels. Their results revealed that the “combined DL model” (Comb.DL.model) significantly out-performed individual clinical and radiomics models, achieving an area under the curve (AUC) of 0.986 in the training set and 0.965 in the testing set, demonstrating its superior diagnostic performance in this challenging clinical context [73].

### 2.6. Performance of AI-Based Detection in PCa

AI-based methods for detecting PCa, specifically identifying cancer-suspicious areas in prostate MRI scans, have called attention of researchers. Despite issues arising regarding their generalizability and clinical applicability, these approaches have shown strong potential to enhance diagnostic accuracy and efficiency. A systematic review of 12 studies evaluating the diagnostic accuracy of ML models for detecting csPCa found that models using biopsy as the reference standard had a pooled AUC of 0.85 (95% CI: 0.79–0.91), while those using radical prostatectomy specimens showed a pooled AUC of 0.88 (95% CI: 0.76–0.99). However, subgroup analysis indicated that non-DL methods outperformed DL-based models (pooled AUC of 0.90 vs. 0.78) [75]. Similarly, Michaely et al. reviewed 29 studies comparing ML and DL techniques on interpreting bpMRI scans, finding no clear performance advantage between the two approaches. While many studies still required manual radiologist input, detection rates and tumor identification using AI techniques were comparable to those of trained radiologists [68].

A systematic review by Alqahtani [76] further highlighted significant contributions to the field. For instance, Yu et al. developed a DL-based AI-assisted PI-RADS system that outperformed 70% of radiologists in MRI-based PCa diagnosis [77]. Hosseinzadeh et al. demonstrated 87% sensitivity for PI-RADS ≥ 4 lesions [78], and Khosravi et al. developed an AI-based model for the early detection of PCa using MR images in a retrospective study which employed CNN for AI-aided biopsy, achieved AUCs of 0.89 for distinguishing between cancerous and benign cases, and 0.78 for differentiating high-risk from low-risk prostate disease. By integrating biopsy report data with MR images, the trained model enhanced prediction accuracy beyond the capabilities of MR images alone [79]. In the mentioned review by Alqahtani et al., it is highlighted that the studies reviewed demonstrate enhanced diagnostic accuracy and moderate sensitivity in PCa, with performance varying according to factors such as the quality of training data and lesion characteristics, including PI-RADS scores. The findings suggest that AI holds substantial promise for improving PCa diagnosis, especially when employed as a second opinion tool for MRI interpretations [76].

Further advancements were reported by Winkel et al., who evaluated the effect of AI on interpreting bpMRI scans in 100 patients. Incorporating AI improved the radiologists’ accuracy in detecting lesions, reflected by an AUC increase from 0.84 to 0.88. Additionally, the inter-reader agreement (measured by Fleiss’ kappa ($\kappa_{F}$)) improved from 0.22 without AI to 0.36 with AI, while reading times were reduced by 21% [80].

A recent study evaluated the performance of a commercial, fully automated AI software (mdprostate), integrated into the PACS environment, for detecting and grading prostate lesions using bpMRI. In a cohort of 123 patients, the tool achieved an AUC of 0.803 for detecting PCa of any grade. At a PI-RADS ≥4 threshold, it reported 85.5% sensitivity and 63.2% specificity—metrics that align broadly with prior meta-analyses of expert radiologists using PI-RADS v2.1 [81]. However, these results were derived from a retrospective single-center dataset without direct comparison to radiologists on the same cases. Furthermore, the tool demonstrated very low specificity (5.9–7.5%) at lower thresholds (PI-RADS ≥2), raising concerns about false positives in routine screening. While the findings suggest potential for reducing inter-reader variability and standardizing lesion classification, larger, prospective studies with radiologist comparisons and lesion-level analyses are required to confirm clinical utility [81].

Despite promising results, the generalizability of AI models is often constrained by reliance on single-center or single-vendor datasets. To address this limitation, initiatives such as Prostate Imaging: Cancer AI (PI-CAI) [82], Prostate 158 [83], and PROSTATEx [84] have released large-scale, multi-center datasets to support standardized clinical validation. For instance, the international PI-CAI study analyzed over 10,000 MRI examinations and found that an AI system achieved an AUROC of 0.91 in detecting Gleason grade group 2 or higher cancers, compared to 0.86 for a pool of 62 radiologists under controlled reading conditions [22]. While the AI system showed improved sensitivity and a reduction in false positives within this retrospective, reader-based evaluation, it did not achieve non-inferiority compared to routine clinical reports in real-world multidisciplinary practice. These findings underscore AI’s potential as a supportive diagnostic tool but also highlight the need for prospective validation before it can be reliably translated into clinical workflows.

In another study, a fully automatic DL system was evaluated for detecting and segmenting csPCa using prostate MRI from two external institutions. The system showed comparable performance across datasets, with AUROC values of 0.80, 0.87, and 0.82. These results suggest that AI models can generalize across different institutions without retraining, although fine-tuning of probability thresholds is necessary for optimal performance [85].

Zhao et al. developed DL models using multicenter bpMRI data from seven hospitals to detect csPCa. Their integrated model, PIDL-CS, demonstrated an AUC of up to 0.914 and improved specificity compared to PI-RADS in some validation cohorts [86]. However, the study was retrospective, with no direct comparison between radiologists and AI on the same cases, and diagnostic thresholds were model-specific and sensitive to tuning. Performance varied across centers, and specificity gains were not universal—PIDL-CS showed lower sensitivity or specificity in some cohorts. Moreover, many patients did not undergo radical prostatectomy, and ground truth often relied on biopsy findings, limiting pathological certainty. These findings underscore AI’s potential but highlight the need for prospective validation and standardized evaluation protocols before clinical adoption.

Karagoz et al. evaluated a self-adapting deep network for detecting csPCa using large-scale bpMRI data from multiple centers. The model, trained on PI-CAI dataset (1500 scans), achieved an AUROC of 0.888 and 0.889 on external validation data. Performance on in-house data were also strong (AUROC 0.886), with a slight decrease to 0.870 using transfer learning. These results highlight the model’s effectiveness and generalizability across diverse datasets, demonstrating the potential of large-scale DL for improving PCa detection [87]. In a very recent study, a self-supervised learning method was integrated into a newly developed transformer-based model to enhance network generalizability in PCa detection. The model demonstrated high performance on external datasets, further improving network generalization [88].

Lastly, a recent meta-analysis by Molière et al. evaluated DL models for detecting and characterizing csPCa across 25 studies. Reported AI performance varied considerably, with AUROC values ranging from 0.573 to 0.892 at the lesion level and 0.820 to 0.875 at the patient level [89]. Comparative studies showed significant heterogeneity between AI and radiologist performance, reflecting differences in datasets, tasks, and validation methods. In several studies, AI sensitivity approximated that of experienced radiologists, while specificity was generally lower, indicating a higher rate of false positives—particularly for PI-RADS 3 lesions [89]. Some studies, such as Seetharaman et al., reported that AI slightly underperformed in sensitivity compared to experts but exceeded the performance of less experienced radiologists [90]. Others, like Zhao et al., demonstrated high AI sensitivity (up to 98.6%) and diagnostic accuracy similar to that of radiologists [86]. These findings underscore AI’s potential but also highlight the variability and need for standardization and prospective validation across diverse clinical environments.

In summary, while AI-based models for PCa detection on MRI show strong potential in improving diagnostic accuracy and efficiency, challenges remain. Variability in study methodologies and data sources highlight the need for standardized, multicentric research and transparent reporting to ensure these models’ robustness and generalizability in clinical settings. A brief comparison of performance metrics is presented in Table 2.

## 3. Challenges and Future Directions

### 3.1. Challenges in AI-Based Quality Control of Prostate MRI Acquisition

Deploying AI models in clinical settings requires careful planning. The current state of AI in prostate MRI quality control and enhancement remains in its early stages, with only a limited number of studies demonstrating the feasibility of AI methods. DL-based reconstruction algorithms have been proposed to accelerate MRI acquisition, particularly for T2WI and DWI. Specifically, studies focusing on T2WI sequences have been restricted to particular modalities or a single imaging plane, which may result in volume averaging and hinder the visual assessment of anatomical structures [91].

Research on AI-based quality evaluation of prostate MRI is still limited. While DL techniques have shown promise in improving or maintaining diagnostic performance in prostate MRI quality, there is insufficient evidence to support their clinical applicability [33,35,92,93]. A primary reason for this is that most methods have been developed and tested using images from the same center, scanner, or protocol, which can lead to inconsistent performance across different distributions [35,92,94]. Additionally, the methods developed for image quality evaluation, such as the PI-QUAL scoring system, include subjective components, such as considerations of anatomical landmarks and artifacts.

Furthermore, the PI-QUAL scoring system is a newly developed quality control framework, and the PI-QUAL criteria have been simplified in a recently published updated version. In the new version of the PI-QUAL scoring system, 10 criteria have been defined, including the ability to clearly delineate the relevant structures in the prostate, the assessment of prevalent artifacts, and the evaluation of image degradations that severely affect the prostate for each sequence individually [19]. However, moderate agreement among readers with varying levels of experience is reported in a recent study that investigates the interobserver variability of PI-QUAL V2. This implies the necessity of further studies to evaluate the clinical applicability and effectiveness of PI-QUAL V2 [47]. In conclusion, the improvement, standardization, and assurance of prostate MRI image quality is still open to the contribution of DL-based methods which are themselves in the research phase and are open to further development.

### 3.2. Challenges and Standards in AI-Based Prostate MRI Interpretation

In addition to the need for high-quality prostate MRI data, selecting the appropriate cohort and ensuring accurate segmentation are critical yet challenging steps for training and testing AI models. Key factors essential for the successful development of AI models in prostate MRI interpretation include using relevant cohorts, digital tumor annotation, proper histopathological correlation, and accurate tumor mapping for MRI verification. However, the entire process of developing a trained model is time-consuming and requires experienced readers. Furthermore, the issue of experience adds another challenge, as the findings of readers performing PCa annotations on prostate MRI images are subjective and strongly influenced by their level of expertise. This subjectivity leads to variations in interpretation, which further complicates the AI model’s reliability.

Due to the subjectivity in PCa annotation, lesion contouring is often performed under supervision to ensure consistency and reliability. Lesion contouring is typically performed by a junior radiologist or non-medical investigators based on clinical radiology reports, under the supervision of an experienced uroradiologist. Fewer studies involved annotations made by two or more readers in consensus [95,96,97]. A recent study has been conducted in which lesion contouring was performed by multiple independent radiologists to account for inter-reader variability [86]. Furthermore, a few recent studies employed a multidisciplinary approach to lesion contouring, involving both a radiologist and a pathologist [98,99]. This approach helps to reduce inter-reader variability and improve the accuracy of tumor delineation.

Recent AI technologies using DL techniques carry the risk of overfitting. To mitigate bias, large population cohorts are essential. In a recent systematic review, primarily focusing on contemporary advancements utilizing large public datasets such as PROSTATEx and PI-CAI, 25 studies were analyzed. It was found that private databases were unicentric in ten studies and multicentric in nine studies, with up to nine centers involved. The training datasets from these private databases ranged in size from 78 to 2170 patients (median: 637, mean: 724). The testing dataset sizes ranged from 41 to 1002 patients (median: 333, mean: 365). The small size and limited diversity of some cohorts can lead to biases that affect the model’s generalizability [89].

Given the challenges in cohort selection and standardization, recent reviews have aimed to establish best practices for AI model development. Turkbey and colleagues have published a comprehensive review addressing the standards and challenges associated with cohort selection in the context of AI model development. According to this review, the reference standard derived from histopathological diagnosis is of critical importance. Ideally, the AI model for prostate MRI interpretation should be trained and tested using verified cases of both PCa and non-PCa. Turkbey et al. proposed the combination of MRI-targeted biopsy, radical prostatectomy, and systematic TRUS-guided biopsy for histopathological verification in the training and testing of AI models. In this approach, systematic biopsies are used to confirm the absence of PCa [65]. This strategy reduces selection bias by mitigating the limitation of radical prostatectomy, which excludes low-risk PCa patients, as well as the limitation of MRI-targeted biopsy, which is limited to true-positive and false-negative cases, leaving no opportunity to assess the false positives identified by the AI model. In a recent systematic review by Molière et al., the ground truths used in AI-based models were examined across 25 studies [89]. According to the review, some studies (approximately 20%) used both systematic and targeted biopsies, guided by transrectal ultrasound, as the ground truth. In other studies (16%), only targeted biopsies with transperitoneal MRI guidance were utilized. A group of studies (24%) relied solely on pathological data from targeted biopsies. Around 32% of the studies used radical prostatectomy as the ground truth, either for the entire population or for selected cases. In the remaining studies (8%), different ground truths were applied to different subcohorts, such as using imaging-based ground truth for the training cohort and biopsies for the testing cohort, or a combination of radical prostatectomy and biopsies. In addition, unlike previous studies, some recent studies have included cases without PCa. In these studies, normal cases were defined as participants without PCa lesions in MRI (PI-RADS ≤ 2 [85,86,87,100,101] or PI-RADS ≤ 3 [102]) and without csPCa in systematic biopsies.

The variability in methods, data, and result reporting among studies has led to concerns about the reproducibility of findings. To address this, radiology AI experts introduced the checklist for artificial intelligence in medical imaging (CLAIM) checklist, modeled on standards for reporting of diagnostic accuracy studies (STARD) guidelines [103], to ensure transparent and reproducible reporting in AI-based medical imaging studies [104]. This initiative has contributed to improvements in the consistency of AI research in prostate MRI. To evaluate the adherence to the CLAIM checklist in studies, a systematic meta-analysis was conducted. The majority of studies provide comprehensive details on model descriptions, training methods, ground truth definitions, data partitioning, and performance metrics. However, information about the study population is reported less consistently, with fewer studies detailing eligibility criteria, demographic and clinical characteristics, or providing participant flowcharts. While the ground truth reference standard is widely described, specifics regarding the source of annotations and the qualifications of annotators are less frequently included. Furthermore, the tools used for annotation, as well as assessments of inter- and intra-reader variability, are infrequently reported. External validation, ensembling techniques, and failure analyses are also addressed in a relatively small proportion of studies.

Another essential aspect of validation is the comprehensive reporting of performance metrics. All relevant metrics, including sensitivity, specificity, PPV, negative predictive value (NPV), and the frequency of false-positive results per patient, should be thoroughly disclosed. In line with this emphasis, these metrics are dominantly reported in recent studies.

Most AI developments in prostate image interpretation have focused on bpMRI, which typically includes T2WI and DWI, often emphasizing axial plane imaging. All models incorporate the T2WI sequence as an input. A significant number of models use bpMRI, with various combinations of T2W, ADC, and DWI sequences, while some models rely solely on T2WI and ADC. A smaller subset of models utilize mpMRI, which adds DCE-MRI to the standard sequences. Additionally, certain networks incorporate zonal segmentations, while others follow a more comprehensive approach and integrate additional information, such as lesion location or histopathological whole slice data, to enhance model performance.

The local prevalence and anatomical characteristics of PCa in different populations affect the performance of AI systems. As a result, the generalizability of AI models is limited, as most existing prostate MRI datasets focus on specific patient populations. Consequently, AI systems for PCa are typically trained on datasets that are optimized for the disease prevalence in particular populations. In other words, AI models trained on such data may not perform well when applied to populations with different disease prevalences or varying definitions of PCa. For instance, according to Penzkofer et al., the benefits of MRI have been most apparent in Western populations, where secondary screening for PCa shows a prevalence of grades 2–5 (according to the International Society of Urological Pathology (ISUP) system) ranging from 30 to 50% [105]. Key challenges in AI based MRI interpretation are presented in Table 3.

## 4. Discussion

The integration of AI in csPCa detection, MRI quality control, and interpretation has shown considerable promise, but challenges remain that must be addressed to fully realize its potential in clinical settings. Across the three areas of focus—AI-based detection of csPCa, AI in MRI quality control, and the interpretation of prostate MRI images using AI—the benefits are evident, but several issues persist that may limit the applicability of these technologies in routine clinical practice. AI-based methods for detecting csPCa, particularly through prostate MRI scans, have demonstrated strong diagnostic potential.

Various studies and systematic reviews suggest that AI models may achieve diagnostic accuracy comparable to that of radiologists, with some reporting marginal performance advantages in specific tasks such as detecting high-risk lesions. However, these results are often based on retrospective analyses, and performance varies notably depending on the dataset, clinical context, and evaluation criteria. Robust prospective validation across diverse clinical settings is needed to confirm the reliability and generalizability of these findings.

For instance, the performance of an AI system developed for the detection of PCa with a Gleason grade group of 2 or higher was compared to that of 62 radiologists (45 centers, 20 countries). The AI system was found to be statistically superior, with an AUROC of 0.91 compared to the radiologists’ AUROC of 0.86 (0.83–0.89). The AI system reduced false-positive results by 50.4%, identified 20% fewer indolent cancers, and maintained the same detection rate for csPCa [22]. While the PI-CAI study offers compelling evidence for AI’s potential, it was retrospective and involved radiologists reading under controlled conditions rather than real-world clinical workflows. Notably, the AI system did not achieve non-inferiority compared to standard-of-care radiology reports, and its development on predominantly European datasets may limit generalizability. These promising findings still require prospective validation before clinical adoption.

Additionally, a meta-analysis involving 25 studies evaluated the performance of DL models for detecting csPCa. The AUROC for lesion-level detection ranged from 0.573 to 0.892, while at the patient level, it ranged from 0.82 to 0.875. When comparing AI to radiologists, AI’s sensitivity was generally comparable to or similar to that of experienced radiologists, and it performed better than junior radiologists. However, the specificity of AI was slightly lower than that of radiologists, particularly with a higher false-positive rate for PI-RADS 3 lesions. This may result in an increased number of unnecessary biopsies, subsequently leading to higher costs, patient anxiety, and potential procedural harm [89].

Despite these promising findings, there are ongoing concerns regarding the generalizability and applicability of AI models across different clinical settings. Many studies have been conducted in single-center environments or have used data from a single imaging protocol, which may limit the ability to apply these models in broader, multi-center clinical practice. Furthermore, variations in lesion characteristics (such as PI-RADS scores) and the quality of training data significantly affect AI performance. While multi-center datasets such as PI-CAI and PROSTATEx are helping to address this limitation, further efforts are needed to improve the robustness and adaptability of AI models across diverse patient populations and healthcare settings [75,76,80,81].

As these challenges are addressed through improved dataset diversity, standardized reporting guidelines, and more rigorous external validation studies, AI has the potential to transform PCa diagnosis. However, its clinical implementation must be approached cautiously, with ongoing monitoring of false-positive rates, cost-effectiveness, and real-world impact on patient outcomes. Achieving this balance will be essential to ensuring that AI serves as a reliable decision-support tool rather than an additional source of diagnostic uncertainty [22,65,89,104].

The potential of AI to improve the quality of prostate MRI acquisition is an exciting development, especially in the context of image reconstruction and acceleration of acquisition times. DL-based reconstruction algorithms have shown the ability to enhance image quality by addressing issues like volume averaging and minimizing visual degradation, which can hinder accurate diagnosis. However, while AI has demonstrated promise in improving MRI quality, it remains in the early stages of clinical adoption, and challenges persist.

One of the key hurdles is that many studies evaluating AI for MRI quality control have been conducted in single-center settings with specific protocols, limiting the generalizability of these findings to other institutions with different imaging practices. Moreover, the development of standardized methods for evaluating image quality remains a challenge. The PI-QUAL scoring system, for example, provides a framework for assessing image quality, but its subjective nature and moderate interobserver variability highlight the need for further refinement. AI technologies hold the potential to standardize quality assessments, but more research is needed to determine their real-world clinical applicability.

Overcoming these hurdles will be essential if AI is to play a meaningful role in routine quality control for prostate MRI [33,35,47,92,93]. The application of AI in prostate MRI interpretation brings unique challenges, particularly in the areas of cohort selection, image annotation, and lesion delineation. The accuracy and reliability of AI models are closely linked to the quality of the data used for training and testing. Variability in lesion annotations, often influenced by the experience level of the annotators, can introduce bias into AI models. Additionally, while some studies have attempted to mitigate this issue by employing multiple annotators or a multidisciplinary approach, the subjectivity in lesion delineation remains a persistent challenge. Inter-reader variability, even among experienced radiologists, further complicates the task of developing reliable and consistent AI models.

In response to this challenge, several recent studies have advocated for the use of large, diverse cohorts to minimize bias and improve the generalizability of AI models. Multi-center datasets and collaborative efforts like PROSTATEx and PI-CAI have shown that AI can be trained on diverse data sources, resulting in models that demonstrate robust performance across different institutions and patient populations. However, as AI models are increasingly tested across multi-center datasets, the risk of overfitting and the potential for performance variability must be carefully managed.

Additionally, achieving transparency and consistency in AI reporting, such as through the CLAIM checklist, is essential for ensuring that AI models are developed and evaluated according to high standards. Moreover, variability in the local prevalence of PCa and the anatomical characteristics of the disease in different populations affects AI model performance. AI systems trained on datasets optimized for specific disease prevalences may face difficulties when applied to populations with different characteristics. This issue highlights the importance of diversity in training datasets to improve the generalizability of AI models in clinical practice [86,87,89,103].

## 5. Conclusions

### 5.1. Main Findings

This narrative review provides a comprehensive synthesis of AI applications in MRI-guided PCa diagnosis, particularly in the pre-biopsy setting. The review integrates and contextualizes developments across three core domains: (1) standardizing and improving MRI quality; (2) enhancing the detection of csPCa; and (3) reducing inter-reader variability.

AI-based models have demonstrated diagnostic accuracy generally comparable to that of radiologists in retrospective studies. A large multicenter reader study involving 62 radiologists from 45 centers across 20 countries reported that an AI system achieved an AUROC of 0.91, exceeding the pooled radiologist performance of 0.86 [22]. Nevertheless, these findings were observed under controlled conditions; in real-world comparisons with standard-of-care reports, non-inferiority was not established.

Meta-analyses underscore substantial variability in AI diagnostic performance, with AUROC values ranging from 0.573 to 0.892 at the lesion level and 0.82 to 0.875 at the patient level [89]. Sensitivity of AI systems often matches that of expert radiologists, though specificity tends to be lower, particularly in PI-RADS 3 lesions.

Beyond lesion detection, AI has shown promise in MRI quality control and acquisition acceleration. DL-based image reconstruction has improved SNR, reduced scan times, and enhanced lesion detectability, especially in high b-value DWI sequences [33,35,47,92,93].

AI also holds value in radiologist education and training by providing standardized feedback and objective evaluation frameworks. In this sense, AI is positioned not as a replacement for radiologists, but as an augmentative tool to support consistency, reproducibility, and diagnostic confidence.

### 5.2. Limitations and Biases

Despite its promise, AI integration into clinical prostate MRI workflows faces several limitations.

First, the vast majority of studies are retrospective in design and based on single-center or single-vendor datasets. This limits the generalizability of findings and may introduce biases related to patient population, scanner type, and imaging protocol. Standardized comparisons of AI models are further hindered by inconsistent reporting of technical details such as model architecture, computational efficiency, and integration within clinical systems.

Many studies rely on biopsy-proven pathology rather than whole-mount prostatectomy, which may lead to under- or overestimation of diagnostic accuracy. Annotation variability and inter-reader inconsistency also remain unresolved issues, particularly affecting lesion localization tasks.

Furthermore, AI performance is context-dependent: high sensitivity may come at the cost of lower specificity, especially for borderline lesions such as PI-RADS 3. This can increase false-positive findings and potentially lead to unnecessary biopsies or overtreatment, thus undermining AI’s potential benefit.

Although AI models have demonstrated promising diagnostic accuracy, most studies lack comparative analysis on computational efficiency, such as inference speed, hardware dependencies, and scalability across institutional infrastructures. Moreover, limited evidence exists on how seamlessly AI tools integrate into routine clinical workflows, including compatibility with PACS/RIS systems, radiologist usability, and interoperability challenges. Future work should benchmark AI tools not only on diagnostic performance but also on operational feasibility metrics relevant to healthcare IT settings.

### 5.3. Future Directions

Moving forward, robust prospective validation of AI systems across diverse patient populations and institutional settings is essential. Studies should be designed to assess not only diagnostic accuracy, but also AI’s impact on clinical workflows, inter-reader agreement, patient outcomes, and healthcare system efficiency.

Future studies should also incorporate operational benchmarks—such as inference latency, integration cost, interface usability, and radiologist workflow impact—into their evaluation protocols to better assess clinical deployability.

Future research must prioritize multicenter collaborations utilizing harmonized MRI protocols, high-quality lesion annotations, and histopathology-confirmed reference standards. Large-scale benchmarking efforts—such as PI-CAI, PROSTATEx, and Prostate158—play a critical role in establishing external validity and mitigating bias.

Standardized evaluation frameworks like the CLAIM checklist and PI-QUAL criteria should be routinely applied to ensure transparent reporting and replicability. Integration into PACS and radiology information systems, with human–AI interaction designed for clinical usability, will be key to real-world implementation.

Additionally, ensuring model explainability, performance monitoring, and regulatory compliance will be essential to gain clinical trust. As AI continues to evolve, ethical principles—including data privacy, equity of access, and mitigation of automation bias—must remain central to development and deployment efforts.

Overall, while AI offers substantial opportunities to enhance prostate MRI interpretation, cautious optimism and evidence-based integration must guide its transition from research to clinical practice.

## Figures and Tables

**Table 1:** The minimum sequence parameter criteria recommended by PI-RADS V2.1 for T2-weighted imaging (T2WI), diffusion-weighted imaging (DWI), and dynamic contrast-enhanced (DCE) imaging in prostate MRI are outlined.

**T2-weighted (T2W) Imaging** **TE (Time of Echo):** ≤90 ms **TR (Time of Repetition):** ≥3000 ms **Slice Thickness:** ≤3 mm **Gap:** No gap **Field of View (FOV):** 12–20 cm **In-plane Resolution:** ≤0.7 mm (phase) and ≤0.4 mm (frequency) **Fat Saturation:** Recommended ***b*-value:** Not applicable for T2W imaging
**DWI** **TE (Time of Echo):** <5 ms **TR (Time of Repetition):** <100 ms **Slice Thickness:** ≤4 mm **Gap:** No gap **Field of View (FOV):** 16–22 cm (encompassing the entire prostate gland and seminal vesicles) **In-plane Resolution:** ≤2.5 mm (phase and frequency) **Fat Saturation:** Recommended (also perform subtraction) ***b*-value**: ○ At least two *b*-values (0–100 s/mm^2^ and 800–1000 s/mm^2^) to calculate apparent diffusion coefficient (ADC) map ○ High *b*-value ≥1400 s/mm^2^ **Temporal Resolution:** ≤15 s **Total Observation Time:** ≥2 min
**DCE Imaging** **TE (Time of Echo):** <5 ms **TR (Time of Repetition):** <100 ms **Slice Thickness:** 3 mm **Gap:** No gap **Field of View (FOV):** 16–22 cm (encompassing the entire prostate gland and seminal vesicles) **In-plane Resolution:** ≤2 mm (phase and frequency) **Fat Saturation:** Recommended ***b*-value:** Not applicable for DCE imaging **Dose and Injection Rate:** ○ **0.1 mmol/kg** of gadolinium-based contrast agent (GBCA) ○ **2–3 cc/s** injection rate, starting with continuous image data

**Table 2:** The findings and performance metrics of various studies evaluating AI-based PCa detection methods. Diagnostic accuracy, including area under the curve (AUC) values, sensitivity, and specificity, across multiple AI models and studies are presented. AI’s diagnostic accuracy was generally comparable to experienced radiologists in various retrospective studies, though results varied considerably due to differences in study designs, populations, and clinical settings. The table also illustrates the variability in AI performance across different study designs, datasets, and clinical settings. The studies reflect both the potential and the challenges of integrating AI into PCa diagnosis.

Study/Technology	Findings	AUC/Performance Metrics
Cuocolo et al. (12 Studies) [75]	Non-DL models outperformed DL models (AUC = 0.90 vs. 0.78).	Machine learning (ML) models using biopsy as reference: AUC = 0.85; radical prostatectomy specimens: AUC = 0.88.
Michaely et al. (29 Studies) [68]	No clear performance advantage between ML and DL methods; AI detection comparable to trained radiologists.	AUC values across studies ranged widely from 0.70 to 0.99, with some studies not providing AUC values.
Yu et al. (DL-assisted PI-RADS) [77]	Outperformed 70% of radiologists in MRI-based PCa diagnosis.	AUC not specified, but outperformed radiologists.
Hosseinzadeh et al. (Patient-Based * Results) [78]	DL-Computer aided diagnosis (DL-CAD) trained with larger sets (1586 scans) performed significantly better. The inclusion of zonal segmentations as prior knowledge improved performance.	AUC: 0.85 (with zonal segmentation and largest training set); Sensitivity: 91% (PI-RADS); Specificity: 77% (PI-RADS); Cohen′s kappa (*κ_c_*) Agreement: 0.53 (DL-CAD vs. radiologists), 0.50 (DL-CAD vs. pathologists), 0.61 (radiologists vs. pathologists).
Hosseinzadeh et al. (Lesion-Based ** Results) [78]	Larger training sets (50–1586 cases) improved performance. Adding zonal segmentation increased sensitivity at similar FP levels.	Sensitivity at 1 FP per patient: 83% (without zonal segmentation), 87% (with zonal segmentation) (95% CI: 82–91); free-response receiver operating characteristic (FROC) Curve Sensitivity: 85% (DL-CAD, with zonal segmentation, 95% CI: 77–83) at 1 FP per patient, compared to expert radiologists’ 91% (95% CI: 84–96) at 0.30 FP per patient.
Khosravi et al. (AI-aided biopsy model) [79]	AUC of 0.89 for distinguishing cancerous vs. benign, AUC of 0.78 for high-risk vs. low-risk disease.	AUC: 0.89 (cancerous vs. benign), 0.78 (high-risk vs. low-risk).
Winkel et al. (AI impact on bpMRI in 100 patients) [80]	AI improved radiologists’ accuracy in detecting lesions, AUC increased from 0.84 to 0.88; Inter-reader agreement improved from 0.22 to 0.36; 21% reduction in reading times.	AUC: 0.88, Fleiss′ kappa (*κ_F_*): improved from 0.22 to 0.36, reduced reading time by 21%.
Bayerl et al. (mdprostate-Commercial AI tool integrated into picture archiving and communication system (PACS)) [81]	100% sensitivity at PI-RADS ≥ 2 cutoff, 85.5% sensitivity, 63.2% specificity at PI-RADS ≥ 4 cutoff; AUC of 0.803 for cancers of any grade.	Sensitivity: 100% (PI-RADS ≥ 2), 85.5% (PI-RADS ≥ 4); Specificity: 63.2%, AUC: 0.803.
Saha et al. (Prostate imaging: Cancer AI (PI-CAI) Study 10,000+ MRI exams) [22]	“Gleason grade group 2 or higher PCa, reduced false positives by 50.4%, detected fewer indolent cancers, improved patient outcomes.	Area under the receiver operating characteristic curve (AUROC): 0.91 (AI vs. 0.86 radiologists).
Netzer et al. (DL system from 2 external institutions) [85]	Comparable performance across external datasets, with receiver operating characteristic (ROC) AUC values of 0.80, 0.87, 0.82.	ROC AUC: 0.80, 0.87, 0.82.
Zhao et al. (Multicenter bpMRI from 7 centers) [86]	DL models showed comparable performance to expert radiologists’ PI-RADS assessment; integration with PI-RADS increased specificity.	Comparable to expert radiologists, increased specificity with PI-RADS integration.
Karagoz et al. (PI-CAI dataset) [87]	AUC of 0.888 and 0.889 on external validation; AUC of 0.870 using transfer learning.	AUROC: 0.888, 0.889 (external validation), 0.870 (transfer learning).
Li et al. (self-supervised learning-transformer-based model) [88]	High performance on external datasets, improving network generalization.	Cross-validation (PI-CAI dataset): AUC: 0.888 ± 0.010, Average Precision (AP): 0.545 ± 0.060; External dataset (model generalizability): AUC: 0.79, AP: 0.45.
Molière et al. (Meta-analysis of 25 Studies) [89]	AI performance ranged from AUC of 0.573 to 0.892 at lesion level, 0.82 to 0.875 at patient level; AI sensitivity approached experienced radiologists.	AUC: 0.573–0.892 (lesion level), 0.82–0.875 (patient level); sensitivity comparable to radiologists.
Seetharaman et al. [90]	AI slightly underperformed in sensitivity but outperformed junior radiologists.	AUC: 0.75 (radical prostatectomy), 0.80 (biopsy); AI detected 18% of the lesions missed by radiologists.

* Patient-based results, ** Lesion-based results (underlining is used to differentiate the cases).

**Table 3:** The key challenges faced by AI-based systems in PCa diagnosis.

Lack of Cohort Diversity	Most AI models are developed using data from single centers or specific protocols, leading to inconsistent performance across different distributions.
Subjectivity in Image Quality Evaluation	The evaluation of MRI image quality, such as through the PI-QUAL scoring system, is subjective and dependent on the reader’s experience, leading to variability.
Interobserver Variability	There is moderate agreement among readers with different experience levels, leading to inconsistency in the evaluation of MRI images.
AI Model Overfitting	AI models using DL techniques may suffer from overfitting, especially when trained on small, non-representative datasets.
Dataset Bias and Generalizability	AI models trained on limited or private databases may not generalize well to other populations or settings, reducing their effectiveness.
Limited Experience in AI Model Training	Developing AI models requires extensive training with experienced readers, but the subjective nature of annotation can lead to inconsistent results across different readers.
Standardization of MRI Quality	Standardizing and improving MRI quality for AI models remains challenging, as variations in acquisition methods can impact model performance.
Multidisciplinary Collaboration Challenges	In lesion contouring, collaboration between different specialists (e.g., radiologists, pathologists) is required, but it remains a challenge to ensure consistency.
Clinical Integration and Approval	Broader clinical integration of AI faces challenges in clinical approval, adherence to safety standards, and the need for diverse, large datasets for training.
Data Privacy and Database Limitations	Many AI studies use private or small-scale databases, which limits the applicability and generalization of AI models, especially for larger or multi-center studies.
Training Protocols and Model Updates	AI models need continual updates and improvements to ensure they remain accurate and effective in the clinical setting.
Bias in PCa Detection	AI models may be biased towards the disease prevalence seen in specific populations, limiting their effectiveness in diverse global settings.

## Data Availability

Data requests should be directed to the authors of the publications that are cited in this review.

## Abbreviations

ADC	Apparent diffusion coefficient
AI	Artificial intelligence
AUA	The American Urological Association
AUC	Area under the curve
AUROC	Area under the receiver operating characteristic curve
bpMRI	Bi-parametric MRI
CAD	Computer aided diagnosis
CLAIM	Checklist for artificial intelligence in medical imaging
CNN	Convolutional neural network
csPCa	Clinically significant prostate cancer
DCE	Dynamic contrast-enhanced
DL	Deep learning
DWI	Diffusion-weighted imaging
EAU	European Association of Urology
EPE	Extraprostatic extension
ESUR	The European Society of Urogenital Radiology
FROC	Free-response receiver operating characteristic
GAN	Generative adversarial network
IV	Intravenous
ISUP	International Society of Urological Pathology
ML	Machine learning
mpMRI	Multi-parametric MRI
MRI	Magnetic resonance imaging
NICE	The National Institute for Health and Care Excellence
NPV	Negative predictive value
PACS	Picture archiving and communication system
PCa	Prostate cancer
PI-CAI	Prostate imaging: cancer AI
PI-QUAL	Prostate imaging quality
PI-RADS	Prostate imaging reporting and data system
PPV	Positive predictive value
PRECISION	Prostate evaluation for clinically important disease: sampling using image guidance or not?
PSA	Prostate-specific antigen
PZ	Peripheral zone
RNN	Recurrent neural network
ROC	Receiver operating characteristic
SNR	Signal-to-noise ratio
STARD	Standards for reporting of diagnostic accuracy studies
SVM	Support vector machine
T1WI	T1-weighted imaging
T2WI	T2-weighted imaging
TRUS	Traditional transrectal ultrasound
TSE	Turbo spin echo
